# Factors of patient satisfaction in adult outpatient departments of private wing and regular services in public hospitals of Addis Ababa, Ethiopia: a comparative cross-sectional study

**DOI:** 10.1186/s12913-019-4685-x

**Published:** 2019-11-21

**Authors:** Demiss Mulatu Geberu, Gashaw Andargie Biks, Tsegaye Gebremedhin, Tesfaye Hambisa Mekonnen

**Affiliations:** 1 Department of Health Systems and Policy, Institute of Public Health, College of Medicine and Health Sciences, University of Gondar, P.O. Box 196, Gondar, Ethiopia; 2 Department of Environmental and Occupational Health and Safety, Institute of Public Health, College of Medicine and Health Sciences, University of Gondar, Gondar, Ethiopia

**Keywords:** Patient satisfaction, Private wing, Regular services, OPD, Addis Ababa, Ethiopia

## Abstract

**Background:**

Knowing the factors for patient satisfaction is an important and direct indicator of quality of health care which is essential for providers to fill their gaps. Although few studies have been conducted on patient satisfaction in Ethiopia; but there is limited evidence for comparing patient satisfaction and associated factors in the public and private wing of the health services. Thus, this study aimed to investigate factors of patient satisfaction in adult outpatient departments in the private wing and regular services at public hospitals of Addis Ababa, Ethiopia.

**Methods:**

A comparative institution based cross-sectional study was conducted from March to April 2018. A total of 955 systematically selected patients were interviewed by using an interviewer-administered structured questionnaire. Binary logistic regression analysis was performed. In the multivariable logistic regression analysis *p* value < 0.05 and adjusted odd ratio (AOR) with 95% confidence interval (CI) were used to identify the associated factors.

**Results:**

The overall patient satisfaction was 89.3% (95% CI: 87.2–91.2). At the regular and private wings of outpatient departments it was 88.3% (95% CI: 85.4–91.2) and 90.4% (95% CI: 87.6–93), respectively. At regular service OPD, patient satisfaction was affected by female sex (AOR: 7.78; 95% CI: 2.89–20.93), long waiting time (AOR: 0.22; 95% CI: 0.07–0.73), information on the prevention of recurrent illnesses (AOR: 14.16; 95% CI: 4.58–43.83), and information on drug use and side effects (AOR: 0.22; 95% CI: 0.08–0.63). In private wing, it was affected by being in the age group of 38 to 47 years (AOR: 22.1; 95% CI: 2.39–203.6), attended elementary school (AOR: 4.69; 95% CI: 1.04–21.26), availability of drugs (AOR: 0.14; 95% CI: 0.04–0.58), and the accessibility of latrines (AOR: 6.56; 95% CI: 1.16–37.11).

**Conclusions:**

Patient satisfaction at the private wing and regular adult OPDs’ of public hospitals had no statistically significant difference. Female sex and information on the prevention of recurrent illnesses were factors positively affected patient satisfaction at regular services, whereas at private wing OPDs’ age, attended elementary school, and accessibility of latrines were factors that positively affected patient satisfaction.

## Background

The principle of patient satisfaction is straightforward with the whole health system, and it is also the measurement of health system responsiveness [1, 2]. Even though it is challenging to find an agreed-upon definition, patient satisfaction is a measure of the level of healthcare content they receive from their providers [3, 4]. Patient satisfaction is a result of their expectations and experience after obtaining service from healthcare providers [5, 6]. Additionally, it is expressed through an affective reaction regarding the discrepancy between what the patients expect and what they obtain [7, 8]. With this, if the patients obtained low or weak service than their expectations, then they will be dissatisfied. In other words, if the received service is in line with or beyond patients’ expectations, this will result in patients to be satisfied [8, 9].

Since healthcare organizations are operating in an increasingly competitive environment, patient satisfaction is a crucial indicator of the market share possessed by the healthcare service provider [10]. Patient satisfaction and the performance of healthcare providers are often interrelated events [11, 12]. Therefore, measuring patient satisfaction can help to improve and maintain the quality of service provision [13]. Furthermore, the measurement and knowing about patient satisfaction are crucial to the providers to know their performance status, and it is also important tool for examining and forecasting client expectations [14]. Additionally, nowadays patient satisfaction measurement is integrated with hospital management strategies to monitor quality patient care processes [15, 16]. It is also the direct measurement of organizational strengths and performance of the provision of the services [17].

Patient satisfaction maintains healthcare organizations‘ image, which in turn translated into improved service use and market share [18]. Studies found that patient satisfaction has positive and direct effect on patient trust [13, 19]. This trust can positively affect patients’ perception of their healthcare providers’ knowledge and skill of treatment. On the other hand, this patients’ perception will likely influence their confidence in healthcare providers’ reliability and expertise [19]. Satisfied patients explained their primary healthcare professional as showing authentic interest in their health problems, able to provide clear explanation of the disease and future health fates, gave them plenty opportunities to discuss health as well as how the disease affected their day to day life [13, 20]. Moreover, satisfied patients were more likely to adhere to the appointed dates and the treatment provided by the service providers. In addition to this, they will be motivated to reuse the service of providers and refer this service to other patients [6, 21].

Globally, patient satisfaction ranges from 55% in Mozambique to 99.6% in Kuwait [14, 22–25]. Similarly, in Ethiopia patient satisfaction at the regular services of public hospitals is low in Tigray (43.6%) and high in Addis Ababa (90.1%) [26–34], whereas at the private wing services it is low in Bahir Dar (57.8%) and relatively high in Nekemte (68.84%) [27, 33].

Studies identified a range of factors affecting patient satisfaction such as: socio-demographic characteristics, like age [24, 32, 33, 35–38], sex [27, 39], education [32, 33, 37, 39, 40], occupation [33, 39, 41], and marital status [35, 42] affected patient satisfaction. In addition, convenience, including the availability of services (drugs, ordered laboratory and X-ray in the hospital) and accessibility of services (waiting time, cost of services, transport to the service) [8, 15, 16, 24, 30, 31, 33, 43] were also associated with patient satisfaction. It is also affected by the courtesy of doctors’ explanations of things in understandable ways, looking out of information regarding symptoms, availability of latrines, sign and direction, and drinking water [21, 28, 32, 43, 44].

A private wing is an annex or an extension within a public hospital where medical services are provided to patients through their full coverage of the service payment [45]. In most regions and at the federal level in Ethiopia, public hospitals are allowed to open and operationalize private wings with the primary objectives of improving health worker retention, providing alternatives and choices to private health service users, and generating additional income for health facilities [46]. At the private wing, patients have the opportunity to choose their health personnel and expect to be satisfied by services, but some studies globally showed that patient satisfaction at regular outpatient departments (ROPD) was higher than that of at private wings [22, 29, 47]. Although few studies have been conducted on patient satisfaction in Ethiopia, there is limited evidence for comparing patient satisfaction and associated factors at the two services even though the private wing service is progressing rapidly, which urged us to perform this comparative study. Besides, since monitoring and evaluating this program is essential to check the progress and its worth, policymakers and other researchers will use this research as an input. Thus, this comparative cross-sectional study investigated factors relating to patient satisfaction in the adult outpatient departments of the private and regular services in public hospitals of Addis Ababa, Ethiopia.

## Methods

### Study design and setting

An institution-based comparative cross-sectional study was conducted in Addis Ababa, the capital of Ethiopia. Seven hospitals including, St. Paul’s Millennium Medical College, Menelik II, and Yekatit 12, which comprised private wing and regular services were selected. Regarding human resources there are 2397, 1524, and 832 health professionals at St. Paul’s, Menelik II, and Yekatit 12 hospital, respectively. The hospitals serve over two million population on their 350 beds each for inpatient admissions. Moreover, those hospitals are teaching, referral, and have almost similar wards.

### Source and study population

This study was conducted from March 16 to April 20, 2018. Accordingly, all patients who visited the adult OPDs of Addis Ababa public hospitals for both private wing and regular services in 2018 were the source population, whereas, all patients who went to the adult OPDs of both private wing and regular services in the selected hospitals during the data collection were the study population. Patients who received services simultaneously or at different times and more than once during data collection period, patients who were mentally ill and unable to communicate were excluded.

### Sample size determination and sampling procedure

The sample size was determined by using the double population proportion formula and Epi-info version 7 with an assumptions of 95% confidence level, 80% power (probability of getting a significant result), P1 (proportion of patient satisfaction at regular services) 58.16% and P2 (proportion patient satisfaction at regular services) 68.84% from research done at Nekemte referral hospital [33]. In addition, 1.5 design effect and 5% non-response rate was considered [29, 30, 40]. The final sample size for n1 (for regular service) = 496 and n2 (for private wing) = 496, which yielded a total of 992.

Initially, three hospitals (St. Paul’s Millennium Medical College, Menelik II, and Yekatit 12) were selected by the lottery method, and OPDs in both the regular and private wing services were considered. Then, the calculated sample was allocated proportionally to both private wing and regular services of the selected wards of OPDs by taking the patient flow of the previous year (2017). Finally, the participants were identified by using the systematic random sampling technique.

### Variables and measurements

Patient satisfaction at both the private wing and regular services was the dependent variable. The independent variables were sociodemographic factors (age, sex**,** marital status**,** educational status, family size, occupation**,** and residence); convenience (availability of services, drugs, ordered laboratory and X-ray in hospitals and accessibility of services, waiting time, cost of services, transport to services), communication and relationship (doctors listen carefully, doctors /nurses explain things in understandable ways, information regarding symptoms lookout, and enough time to discuss problems), physical environment/facilities (availability of latrines, signs and directions, and drinking water).

Patient satisfaction was measured using 16 satisfaction measuring items on a five-point Likert scale, together yielded a maximum of 80 and a minimum score of 16. Then the responses to the 16 measuring items were summed and transformed to give an individual level satisfaction score from 1 to 100% for each item. Patients who scored 75% and above on the 16 satisfaction measuring items were satisfied, and those who scored less than 75% were unsatisfied [16, 47, 48].

The patient expectation was measured by the patient’s expectations, wants, and thinks to need to be completed [48].

Getting all services: if the patient perceives, he /she got all the services.

Getting some services: if the patient perceives, he /she got some of the services.

Not getting services: if the patient perceives he /she did not get all of the services.

Private wing services is an annex or an extension within a public hospital where medical services are provided to patients at full cost recovery [45].

Regular services are the services that are given routinely, excluding private wing.

Waiting time is the interval between departure from registration for outpatient service and the moment at which the patient meets the service provider.

Health facility distance was measured in KM, and when the distance of the health facility is located within 10KM from home, it was convenient [33].

### Data collection instruments and procedures

An interviewer-administered structured questionnaire was adapted for data collection after reviewing relevant studies [22, 30, 32, 33, 49]. The questionnaire was first prepared in English (Additional file 1), then translated to Amharic (the predominant local language in Addis Ababa) and was back to English to check its consistency. The questionnaire was translated from English into the local language (Amharic) by the authors with the help of language experts. The back-translation of the Amharic version was performed by senior Academic staffs of the Department of Health System and Policy who were not the member of the research group and not awarded about the original questionnaire. Then, the authors, the language expert and the senior academic staff members were met and discuss the translation and back-translation. Finally, the last Amharic version of the questionnaire was prepared for data collection. The Cronbach’s alpha coefficient for all the satisfaction measurement items was 0.87. Then, data collectors were assigned to the respective sites to conduct exit interviews with the help of ward coordinators.

### Data quality control

Ten data collectors were recruited from accelerated medicine students. Previously, these data collectors were health professionals, at the countryside health facilities of Ethiopia, who have a background of public health and nursing. However, currently they are studying their accelerated medicine education and are not the staff of the three selected hospitals. In addition to the data collectors, three supervisors were recruited to facilitate qualified data collection process. Both data collectors and supervisors were taken a half-day training by the principal investigator about the questionnaire to collect relevant data. The supervisors made close supervision while the principal investigator monitored and facilitated the overall process incognito. A pre-test was conducted on 50 patients (5% of the sample) at Ras Desta Damtew hospital, and necessary corrections and amendments were made on tool before the actual data collection. During data collection, supervisors have checked the data for accuracy, consistency, and completeness in each day of data collection period.

### Data processing and analysis

The completed data were cleaned, coded, and entered to Epi-Data version 3.1 and exported to SPSS version 20 for analysis. Descriptive statistics, text narration, and tables were used to present the results. Binary logistic regression was performed. In the bi-variable logistic regression analysis *p*-value less than 0.2 was used to select the candidate variables for multivariable logistic regression analysis. In the final multivariable logistic regression analysis model, *p* value less than 0.05 and AOR with 95% CI were used to declare the associated factors.

## Results

### Socio-demographic characteristics of the participants

A total of 955 patients answered the questionnaire with a response rate of 96.3%; 488 of the patients with a response rate of 98.4% were from the regular service, and 467 with a response rate of 94.2% were from the private wing of the adult outpatient departments. Nearly fifty-seven and 55 % of the respondents in the regular and private wing services were female, respectively. The median age (with interquartile range) of the respondents in the regular and private wing outpatient departments was 36.0 (IR: 24) and 42.0 (IR: 30) years, respectively. Regarding educational status, 31.4 and 31.3% of the respondents in the regular and private wing outpatient department, diploma and above graduate, respectively. The majority of the respondents 87.7% at the regular and 88.0% at the private wing outpatient departments were urban dwellers, respectively (Additional file 2).

### Type of visits and pre-services

Fifty-two and 53 % of the patients at the regular and private wing OPDs’ were new visitors, respectively. Of these new visitors, 72.2% of respondents at the regular and 68.7% of the private wing patients visited the hospital by their personal decisions. Out of the total respondents, laboratory tests were ordered for 58.4 and 37.5% of patients at regular and private wing OPDs, respectively. X-ray (the other internal organ laboratory) was ordered for 42.0% of the regular service OPD and 34.5% of the private wing patients. Of the total respondents, drug/supplies were ordered for 83.0 and 90.4% of the regular and private wing OPD patients, respectively (Table 1).

**Table 1:** Type of visit and pre-service activities among patients attending ROPD and PWROPD of Addis Ababa public hospitals, May 2018

Variable	ROPD (n = 488)	PWOPD (*n* = 467)	Total (*n* = 955)
	*n* (%)	*n* (%)	*n* (%)
Type of visit			
New	255 (52.3)	249 (53.3)	504 (52.8)
Repeated	233 (47.7)	218 (46.7)	451 (47.2)
How the new respondents visit the hospitals			
With referral	184 (72.2)	171 (68.7)	355 (70.4)
With the recommendation of others	25 (9.8)	29 (11.6)	54 (10.7)
With personal decision	46 (18.0)	49 (19.7)	95 (18.9)
Total	255 (100)	249 (100)	504 (100)
Laboratory was ordered			
Yes	285 (58.4)	175 (37.5)	460 (48.2)
No	203 (41.6)	292 (62.5)	495 (51.8)
X-ray (other internal organ laboratories) was ordered			
Yes	205 (42.0)	161 (34.5)	366 (38.3)
No	283 (58.0)	306 (65.5)	589 (61.7)
Respondents were going to drink water			
Yes	149 (30.5)	77 (16.5)	226 (23.7)
No	339 (69.5)	390 (83.5)	729 (76.3)
Drugs/supplies ordered			
Yes	405 (83.0)	422(90.4)	827 (86.6)
No	83 (17.0)	45(9.6)	1289 (13.4)
Respondents were gone to the toilet			
Yes	293 (60.0)	248 (53.1)	541 (56.6)
No	195 (40.0)	219 (46.9)	414 (43.4)

*ROPD* Regular outpatient department, *PWOPD* Private wing outpatient department

### Perception of respondents on healthcare services availability and accessibility

Drinking water was available for 73.8 and 83.1% of the patients who wanted to drink in the regular and private wing OPDs, respectively. Out of the total respondents, 77.34% and of patients at the regular and 76.9% at the private wing OPDs said that information sign and directions were available. Almost 77 and 74% of patients at the regular and private wing OPD’s, respectively, said that the ordered x-rays were available.

Of patients who wanted to use toilets, 74.8 and 70.2% at the regular and private wing were dissatisfied with the accessibility of latrines, respectively. Forty-one percent of the regular and 36.8% of the private wing patients waited 30 min and less to enter the OPDs. The median waiting time (at the waiting area to see health providers) for both OPDs was 60 min. Out of the total patients who paid for services 86.4%, of the regular and 88.7% of the private wing were satisfied with the service fees (Table 2).

**Table 2:** Availability and accessibility of health care services at ROPD and PWOPD of Addis Ababa public hospitals, May 2018

	ROPD*	PWOPD*	Total
	*n* (%)	*n* (%)	*n* (%)
A. Availability of health services			
Availability of drinking water			
Yes	110 (73.8)	64 (83.1)	174 (77.0)
No	39 (26.2)	13 (16.9)	52 (23.0)
Total	149 (100)	77 (100)	226 (100)
Availability of sign and direction			
Yes	377 (77.3)	359 (76.9)	736 (77.1)
No	111 (22.7)	108 (23.1)	219 (22.9)
Availability of ordered laboratory in the hospital			
All in all	205 (71.9)	102 (58.3)	307 (66.7)
Some	59 (20.7)	48 (27.4)	107 (23.3)
Not at all	21 (7.4)	25 (14.3)	46 (10)
Total	285 (100)	175 (100)	460 (100)
Availability of ordered X-ray (other internal organ laboratories) in the hospital			
All in all	157 (76.6)	119 (73.5)	276 (75.2)
Some	26 (12.7)	22 (13.6)	48 (13.1)
Not at all	22 (10.7)	21 (13)	43 (11.7)
Total	205 (100)	162 (100)	367 (100)
Availability of ordered drugs and supplies in the hospital			
Getting all	216 (53.3)	219 (51.9)	435 (52.6)
Getting Some	158 (39.0)	173 (41)	331 (40.0)
Not getting all	31 (7.7)	30 (7.1)	61 (7.4)
Total	405 (100)	422 (100)	827 (100)
Satisfaction due to the availability of clean latrine			
Dis-satisfied	217 (73.8)	171 (69.0)	388 (71.6)
Satisfied	77 (26.2)	77 (31.0)	154 (28.4)
Total	294 (100)	248 (100)	542 (100)
B. Accessibility of health services			
Travel distance from home to hospital in KM			
< =10	274 (56.1)	256 (54.8)	530 (55.5)
11–40	111 (22.7)	105 (22.5)	216 (22.6)
41–80	27 (5.5)	21 (4.5)	48 (5.0)
> =81	76 (15.6)	85 (18.2)	161 (16.9)
Waiting time to enter OPD (at waiting area) in minute			
< =30	202 (41.4)	172 (36.8)	374 (39.2)
31–60	125 (25.6)	124 (26.6)	249 (26.1)
61–120	83 (17.0)	91 (19.5)	174 (18.2)
121–180	38 (7.8)	37 (7.9)	75 (7.9)
> =181	40 (8.2)	43 (9.2)	83 (8.7)
Satisfaction due to the cost of services			
Dissatisfied	44 (13.6)	47 (11.3)	93 (12.5)
Satisfied	280 (86.4)	369 (88.7)	649 (87.5)
Total	324 (100)	416 (100)	742 (100)
Satisfaction due to the accessibility of latrine			
Dissatisfied	220 (74.8)	174 (70.2)	394 (72.7)
Satisfied	74 (25.2)	74 (29.8)	148 (27.3)
Total	294 (100)	248 (100)	542 (100)

^*^ROPD Regular outpatient department, *PWOPD* Private wing outpatient department

### Information provided by healthcare providers

Almost 58 and 57 % of the respondents at the regular and private wings, respectively, got all in all information on drug uses and their side effects from pharmacy staff. Among the total respondents, 84.2 and 86.1% of regular and private wing patients indicated that health providers told them on prevention of recurrence of illnesses, respectively (Table 3).

**Table 3:** Information provided by healthcare workers and client perceptions of services at ROPD and PWOPD of Addis Ababa public hospitals, May 2018

Information	Response	ROPD* (*n* = 488)		PWOPD* (*n* = 467)	
		Frequency (n)	Percent (%)	Frequency (n)	Percent (%)
The provider told them about the prevention of recurrence of the illness	Yes	411	84.2	402	86.1
	No	77	15.8	65	13.9
Provider interviewed them by the language they can understand	Yes	481	98.6	456	97.6
	No	7	1.4	11	2.4
Information gained on drug use and side effects	Explain all	233	57.5	240	56.9
	Explain some	146	36.0	151	35.8
	Do not explain	26	6.4	31	7.3
	Total	405	100	422	100

^*^ROPD Regular outpatient department, *PWOPD* Private wing outpatient department

### Patient satisfaction

In this study, patient satisfaction was assessed by 16 items of satisfaction measurement. The overall patient satisfaction was 89.3% (95% CI: 87.2–91.2). However, patient satisfaction at ROPD was 88.3% (95% CI: 85.4–91.2) and at PWOPD 90.4% (95% CI: 87.6–93.0).

Of the respondents, 40.2% from the regular and 49.3% from private wing reported that they strongly agreed (strongly satisfied) with the information provided by all other staff (other than doctors and nurses), while 3.9% patients in the regular and 6.1% in the private wing services said that they strongly disagreed with the information. Moreover, 39.1% of the regular and 47.5% of the private wing patients pointed out that they strongly agreed with the length of time spent to get services and get back home, but 4.9% of the former and 4.1% of the private wing patients said that they strongly disagreed with the preceding (Tables 4, 5).

**Table 4:** Level of satisfaction of patients on each satisfaction measuring items with ROPD of healthcare services at Addis Ababa public hospitals, May 2018 (*n* = 488)

Items	Perceived client response at ROPD				
	SDA^a^	DA^b^	Neutral	Agree	SA^c^
	*n* (%)	*n* (%)	*n* (%)	*n* (%)	*n* (%)
Staff behavior and services					
Doctor treats you very friendly and courteous manner	1 (0.2)	5 (1.0)	24 (4.9)	133 (27.3)	325 (66.6)
Doctors are good to explain how to prevent your disease	8 (1.6)	27 (5.5)	39 (8.0)	140 (28.7)	274 (56.1)
Doctors are careful to check everything when treating and examining me	0 (0)	9 (1.8)	26 (5.3)	150 (30.7)	303 (62.1)
How much are you satisfied with the information provided by doctor/nurses (courteous and respectful)	6 (1.2)	18 (3.7)	40 (8.2)	157 (32.2)	267 (54.7)
How much are you satisfied with the information provided by all other staffs (other than doctors and nurses)	30 (6.1)	43 (8.8)	64 (13.1)	155 (31.8)	196 (40.2)
How much are you satisfied with the way health providers listened to you	3 (0.6)	9 (1.8)	18 (3.7)	166 (34.0)	292 (59.8)
How much are you satisfied with measures taken to assure your confidentiality	1 (0.2)	11 (2.3)	33 (6.8)	141 (28.9)	302 (61.9)
How much are you satisfied with the overall quality of health care services in this hospital	6 (1.2)	25 (5.1)	46 (9.4)	179 (36.7)	232 (47.5)
Physical facilities/environment					
Adult OPD location is convenient for you	4 (0.8)	17 (3.5)	29 (5.9)	162 (33.2)	276 (56.6)
How much are you satisfied with the comfortability of chairs in the waiting area	9 (1.8)	28 (5.7)	47 (9.6)	162 (33.2)	242 (49.6)
How much are you satisfied with the cleanness of Waiting area	5 (1.0)	15 (3.1)	38 (7.8)	186 (38.1)	244 (50.0)
How much are you satisfied with the cleanliness of Examination/consultation room /OPD	2 (0.4)	11 (2.3)	34 (7.0)	169 (34.6)	272 (55.7)
How much are you satisfied with the overall cleanliness of the compound	6 (1.2)	19 (3.9)	44 (9.0)	197 (40.4)	222 (45.5)
Accessibility & availability to health care services					
How much are you satisfied with the waiting time to get outpatient services after registration (at waiting area) appropriateness for you	9 (1.8)	37 (7.6)	47 (9.6)	190 (38.9)	205 (42.0)
How much are you satisfied with the time spent to get services and get back (overall waiting time)	24 (4.9)	37 (7.6)	57 (11.7)	179 (36.7)	191 (39.1)
How much are you satisfied with the consultation duration	4 (0.8)	9 (1.8)	23 (4.7)	189 (38.7)	263 (53.9)

*SDA*^a^ Strongly disagree, *DA*^b^ Disagree, *SA*^c^ Strongly Agree

**Table 5:** Level of satisfaction of patients on each satisfaction measuring items at PWOPD of Addis Ababa public hospitals, May 2018 (n = 467)

Items	Perceived client response at PWOPD				
	SDA	DA	Neutral	Agree	SA
	*n* (%)	*n* (%)	*n* (%)	*n* (%)	*n* (%)
Staff behavior and services					
Doctor treats you very friendly and courteous manner	5 (1.1)	4 (0.9)	9 (1.9)	110 (23.6)	339 (72.6)
Doctors are good to explain how to prevent your disease	13 (2.8)	28 (6.0)	34 (7.3)	113 (24.2)	279 (59.7)
Doctors are careful to check everything when treating and examining me	3 (0.6)	3 (0.6)	19 (4.1)	115 (24.6)	327 (70.0)
How much are you satisfied with the information provided by doctor/nurses (courteous and respectful)	5 (1.1)	11 (2.4)	31 (6.6)	120 (25.7)	300 (64.2)
How much are you satisfied with the information provided by all other staffs (other than doctors and nurses)	18 (3.9)	25 (5.4)	65 (13.9)	129 (27.6)	230 (49.3)
How much are you satisfied with the way health providers listened to you	2 (0.4)	11 (2.4)	20 (4.3)	147 (31.5)	287 (61.5)
How much are you satisfied with measures taken to assure your confidentiality	5 (1.1)	16 (3.4)	30 (6.4)	115 (24.6)	301 (64.5)
How much are you satisfied with the overall quality of health care services in this hospital	7 (1.5)	11 (2.4)	37 (7.9)	144 (30.8)	268 (57.4)
Physical facilities/environment					
Adult OPD location is convenient for you	5 (1.1)	16 (3.4)	22 (4.7)	139 (29.8)	285 (61.0)
How much are you satisfied with the comfortability of chairs in the waiting area	5 (1.1)	16 (3.4)	48 (10.3)	134 (28.7)	264 (56.5)
How much are you satisfied with the cleanness of Waiting area	2 (0.4)	12 (2.6)	35 (7.5)	150 (32.1)	268 (57.4)
How much are you satisfied with the cleanliness of Examination/consultation room /OPD	1 (0.2)	10 (2.1)	37 (7.9)	141 (30.2)	278 (59.5)
How much are you satisfied with the overall cleanliness of the compound	4 (0.9)	21 (4.5)	45 (9.6)	144 (30.8)	253 (54.2)
Accessibility & availability to health care services					
How much are you satisfied with the waiting time to get outpatient services after registration (at waiting area) appropriateness for you	5 (1.1)	27 (5.8)	37 (7.9)	156 (33.4)	242 (51.8)
How much are you satisfied with the time spent to get services and get back (overall waiting time)	19 (4.1)	24 (5.1)	44 (9.4)	158 (33.8)	222 (47.5)
How much are you satisfied with the consultation duration	1 (0.2)	10 (2.1)	37 (7.9)	141 (30.2)	278 (59.5)

In our study, patient satisfaction at private wing and regular adult OPD has no statistical significant difference at 95% CI: 0.82–1.88, *p*-value = 0.307 or X^2^ = 1.05, p-value = 0.307.

### Factors associated with patient satisfaction

In the multivariable logistic regression analysis, sex, waiting time, information on prevention of recurrence of illnesses, getting information on the use of drugs and their side effects were significant variables for patient satisfaction in the regular services adult outpatient departments. Female patients were 7.78 times more satisfied (AOR: 7.78; 95% CI: 2.89–20.93) than their counterparts. Patients who waited for 61–120 min to enter OPD were 78% less satisfied (AOR: 0.22; 95% CI: 0.07–0.73) and those who waited for 121–180 min were 87% less satisfied (AOR: 0.13; 95% CI: 0.03–0.62) than patients who waited for 30 min and less. Patients who were informed on the prevention of recurrence of illnesses were 14 times more satisfied (AOR: 14.16; 95% CI: 4.58–43.83) than those who were not informed. Moreover, patients who had got some information on drug use and their side effects were 88% less satisfied (AOR: 0.22; 95% CI: 0.08–0.63) compared to those who had got all in all information (Table 6).

**Table 6:** Bi-variable and multi-variable logistic regression analysis of patient satisfaction at ROPD of Addis Ababa public hospitals, May 2018 (n = 488)

Explanatory Variables	Satisfied n (%)	Dissatisfied *n* (%)	COR (95% CI)	AOR (95% CI)
Sex				
Male	176 (83.0)	36 (17.0)	1	1
Female	255 (92.4)	21 (7.6)	2.48 (1.403–4.398) ^***^	7.78 (2.89–20.93) ^***^
Did the provider told you how to prevent recurrence of the illness				
Yes	381 (92.7)	30 (7.3)	6.86 (3.772–12.47) ^***^	14.16 (4.58–43.83) ^***^
No	50 (64.9)	27 (35.1)		
Drug availability				
All in all	197 (91.2)	19 (8.8)	1	1
Some	132 (83.5)	26 (16.5)	0.49 (0.260–0.921) ^*^	0.68 (0.24–1.92)
Not at all	26 (83.9)	5 (16.1)	0.50 (0.173–1.457)	0.14 (0.02–1.02)
Information gained on drug use and side effects				
Explain all	216 (92.7)	17 (7.3)	1	1
Explain some	116 (79.5)	30 (20.5)	0.30 (0.161–0.575) ^***^	0.22 (0.08–0.63) ^**^
Not explain	26 (89.7)	3 (10.3)	0.60 (0.164–2.215)	2.45 (0.22–26.70)
Waiting time to enter OPD (at waiting area) (in minute)				
< =30	189 (93.6)	13 (6.4)	1	1
31–60	115 (92.0)	10 (8.0)	0.79 (0.34–1.86)	0.62 (0.18–2.11)
61–120	64 (77.1)	19 (22.9)	0.23 (0.11–0.50) ^***^	0.22 (0.07–0.73) ^*^
121–180	30 (78.9)	8 (21.1)	0.26 (0.10–0.68) ^**^	0.13 (0.03–0.62) ^*^
> =181	33 (82.5)	7 (17.5)	0.32 (0.12–0.87) ^*^	0.43 (0.08–2.22)
Satisfaction due to the availability of clean latrine				
Dissatisfied	177 (81.6)	40 (18.4)	1	1
Satisfied	72 (93.5)	5 (6.5)	3.25 (1.235–8.578) ^*^	3.12 (0.233–41.784)
Family size				
1–2	97 (89.0)	12 (11.0)	0.76 (0.34–1.70)	1.67 (0.43–6.48)
3–4	159 (91.4)	15 (8.6)	1	1
5–6	90 (84.9)	16 (15.1)	0.53 (0.25–1.12)	0.39 (0.12–1.27)
> =7	85 (85.9)	14 (14.1)	0.57 (0.26–1.24)	0.34 (0.10–1.17)
Satisfaction due to the availability of clean latrine				
Dissatisfied	180 (81.8)	40 (18.2)	1	1
Satisfied	69 (93.2)	5 (6.8)	3.07 (1.162–8.092) ^*^	0.88 (0.062–12.518)

*P value < 0.05, **P- value <=0.01 ***P- value <= 0.001, *AOR* Adjusted odd ratio, *COR* Crude odd ratio

At the private wing’s adult outpatient departments, age, educational status, availability of ordered drugs and accessibility of latrines were significant variables for patient satisfaction. Patients who were 38–47 years of age were 22 times more satisfied than those who were 48 years and older age group (AOR: 22.04; 95% CI: 2.03–148.15). Patients who attended elementary school (grade 1–8) were 4.69 times more satisfied (AOR: 4.69: 95% CI: 1.04–21.26) than diploma and above graduates in the private wing OPDs. Patients who had got some ordered drugs in the hospital were86% less satisfied than patients who got all the ordered drugs (AOR: 0.14; 95% CI: 0.04–0.58). Patients who were satisfied with the accessibility of latrines were 6.56 times more satisfied (AOR: 6.56; 95% CI: 1.16–37.11) than those of dissatisfied with the latrine services (Table 7).

**Table 7:** Bi-variable and multi-variable logistic regression analysis of patient satisfaction at PWOPD of Addis Ababa public hospitals, May 2018 (n = 467)

Variables	Satisfied *n* (%)	Dissatisfied *n* (%)	COR (95% CI)	AOR (95% CI)
Drug availability				
All in all	211 (96.3)	8 (3.7)	1	1
Some	144 (83.2)	29 (16.8)	0.19 (0.08–0.42) ***	0.14 (0.04–0.58) ^**^
Not at all	27 (90.0)	3 (10.0)	0.34 (0.09–1.37)	13.00 (0.12–145.65)
Age in years				
18–27	77 (87.5)	11 (12.5)	0.84 (0.39–1.83)	2.68 (0.67–10.94)
28–37	87 (91.6)	8 (8.4)	1.305 (0.56–3.07)	2.21 (0.56–8.68)
38–47	83 (94.3)	5 (5.7)	1.99 (0.73–5.47)	22.1 (2.39–203.6) ^**^
> =48	175 (89.3)	21 (10.7)	1	1
Educational status				
Unable to read & write	58 (96.7)	2 (3.3)	5.75 (1.32–25.17) *	10.47 (0.97–113.70)
Able to read and write	43 (89.6)	5 (10.4)	1.71 (0.61–4.75)	3.15 (0.59–16.87)
Grade 1–8	101 (94.4)	6 (5.6)	3.34 (1.31–8.49) ^*^	4.69 (1.04–21.26) ^*^
Grade 9–12	99 (92.5)	8 (7.5)	2.46 (1.11–5.70) ^*^	3.91 (0.95–16.11)
Diploma and above	121 (83.4)	24 (16.6)	1	1
Waiting time to enter OPD (at waiting area) (in minute)				
< =30	161 (93.6)	11 (6.4)	2.37 (0.83–6.83)	2.57 (0.49–13.40)
31–60	115 (92.7)	9 (7.3)	2.07 (0.69–6.21)	1.68 (0.32–8.86)
61–120	78 (85.7)	13 (14.3)	0.97 (0.34–2.76)	1.34 (0.24–7.31)
121–180	31 (83.8)	6 (16.2)	0.84 (0.25–2.86)	1.11 (0.21–6.69)
> =181	37 (86.0)	6 (14.0)	1	1
Information gained on drug use and side effect				
Explain all	228 (95.0)	12 (5.0)	1	1
Explain some	127 (84.1)	24 (15.9)	0.28 (0.14–0.58) ^*^	0.72 (0.22–2.41)
Not Explain	27 (87.1)	4 (12.9)	0.36 (0.11–1.18)	0.13 (0.01–3.69)
Satisfaction due to the accessibility of latrine				
Dissatisfied	141 (81.0)	33 (19.0)	1	1
Satisfied	72 (97.3)	2 (2.7)	8.43 (1.96–36.1) ^***^	6.56 (1.16–37.11) ^*^
Satisfaction due to the cost of services				
Dissatisfied	39 (83.0)	8 (17.0)	1	1
Satisfied	336 (91.1)	33 (8.9)	2.09 (0.90–4.84)	2.77 (0.84–9.12)

*Significant at *P* value < 0.05, **significant at *P* value <=0.01, ***significant at *P* value < 0.001, *AOR* Adjusted odd ratio, *COR* Crude odd ratio, *ROPD* Regular outpatient department, *PWOPD* Private wing outpatient department

Moreover, sex, availability of clean latrine, waiting time, cost of services, information on prevention of recurrence of illnesses and information gained on drug use, and their side effect were factors associated with overall patient satisfaction in the final model of the multivariable logistic regression analysis. Female patients were two times more satisfied (AOR: 2.03; 95% CI: 1.06–3.88) than males patients who were satisfied with the availability of clean latrines were 3 times more satisfied (AOR: 3.34; 95% CI: 1.31–8.50) than who were not; patients who had waited for 61–120 min had 64% decreased satisfaction (AOR: 0.36; 95% CI: 0.15–0.87) when compared with patients who waited for 30 min and less. Patients who were satisfied with the cost of services were 2.46 times more satisfied (AOR: 2.46; 95% CI: 1.10–5.63) than those dissatisfied with costs of services. Patients who were informed on the prevention of recurrence of illnesses were 2.38 times more satisfied (AOR: 2.38; 95% CI: 1.09–5.23) than those who were not informed. Moreover, patients who had got some information on drug use and their side effects had decreased satisfaction by 57% (AOR: 0.43; 95% CI: 0.20–0.90) compared to those who had got all in all information. An additional multivariable logistic regression analysis for overall patient satisfaction file shows more details (Additional file 3).

## Discussion

Overall, 89.3% of patients were satisfied with the services they received. The results of this study showed that 88.3 and 90.4% patients were satisfied with the services they received at the regular and private wing OPDs, respectively. Additionally, overall patient satisfaction was 89.3%. Patient satisfaction with the healthcare service delivery at the private wing and the regular adult OPDs had no statistically differences. This non-difference might be due to the patients‘ expectation of more services for their higher payment in the private wing which reduce satisfaction. That means the patients at private wing service are getting service with high payment than the regular service users, so due to their high payment than the regular service users, they also expect high better services. If they did not obtain compatible service with their expectations, their satisfaction would be lower, even if one objective of the establishment of private wing service was to avail suitable service for those who can afford it. Moreover, in the current study a large proportion of patients in the private wing were older, which might have reduced their patient satisfaction as reported by other studies that as age increased patient satisfaction decreased [29, 33, 38].

In this study, patient satisfaction was higher than those of studies conducted at Nekemt referral hospital both regular and private wing OPDs (58.2 and 68.8%) [33], Hawassa University teaching hospital (80.1%) [29], Jimma University specialized hospital (77.0%) [31], Bahirdar Felegehiwot referral hospital (57.8%) [32], Debrebirhan referral hospital (57.7%) [40], Tigray Zonal hospital (43.6%) [27], Wolaita Sodo University teaching hospital (54.2%) [30], Nigeria University Calabar teaching hospital (59.3%) [25], and Nepal Chitwan Medical College teaching hospital (75.9%) [24]. The difference might be due to the study setup difference. That is, our study was conducted at a more urbanized and capital city of a country with better health infrastructure, transportation access, level of hospital, and different specialized health professionals. Additionally, patients in private wings had chances to choose their best options based on pre-information from other patients. Our finding is comparable with those of studies conducted in Rural Haryana, India (89.1%) [14], and Addis Ababa Black Lion hospital (90.1%) [34]. However, it is lower than the result of a study conducted in the capital health region of Kuwait (99.6%) [22]. This variation might be due to time and study setup differences [26, 38] and variations in the numbers of patients visiting the hospitals. The differences in patient management strategies across the hospitals and the use of different cutoff points to determine patient satisfaction might be the other reason of satisfaction discrepancy. Moreover, this variation might be explained by the difference in socio-economic status. That is; patients from Kuwait are wealthier which helps them to access more health services as per their need.

Our findings showed that female patients were 7.78 times more satisfied than males in ROPDs. In agreement with this study, a critical review of determinants’ of patient satisfaction with healthcare system in Pakistan reported that female sex was associated with good satisfaction [38], and another study conducted in another year in Pakistan also indicated that being female was associated with lower likelihood of being dissatisfied [39]. Additionally, a study conducted in Addis Ababa public hospitals, Ethiopia, found that female patients (74%) were more satisfied than male patients (69%) [26]. In contrast, females were found to be less satisfied than males in a study conducted at primary healthcare services in the capital health region, Kuwait [22]. This difference might be related to cultural variations.

Regarding the accessibility of healthcare services at the regular OPDs and in the overall model of patient satisfaction, waiting time was a statistically significant variable. Patients who had waited 61–120 min had their satisfaction decreased by 78% and those who had waited 121–180 min by 87% compared with patients who had waited 30 min and less. Comparably, in the overall model of patient satisfaction, patients who had waited 61–120 min had decreased their satisfaction by 64% compared with patients who waited 30 min and less. This finding was supported by studies at Debrebirhan referral hospital [40], Jimma hospital [44], and Wolaita Sodo University teaching hospital [30].

The study revealed that patients who informed on prevention of recurrence of illness were 14 and 2.38 times more satisfied than those who were not informed in ROPD and the overall model of patient satisfaction, respectively. This finding is comparable with that of a study done in Wolaita Sodo University teaching hospital [30]. Patients who were got some information on drug use and their side effects were 78 and 57% less satisfied than those who were got all the information at ROPD and in the overall model of patient satisfaction, respectively. This result is in contrast with a study finding in the Nekemt referral hospital and revealed that 89.3% of patients were satisfied by health providers’ information on drug use and their side effects [33]. This difference might be due to the high patient load at Addis Ababa public hospitals, especially in ROPD resulting in shortage of time for the healthcare providers to explain everything to their patients.

In the private wing outpatient departments, patients who were in the age category of 38–47 years were 22 times more satisfied than those 48 years and older age groups. This finding is consistent with that of a study conducted at Bahir Dar Felege Hiwot referral hospital at Private wing services showed that 37–47 and greater than 48 years of age decreased satisfaction by 53 and 60%, respectively, compared with 18–27 years of age [32]. This lower satisfaction might be because as age increases, expectations rise; besides, enhanced knowledge and experience decrease satisfaction [40, 44]. Nevertheless, in contrast to this study, a study in Jimma hospital reported that the proportion of users satisfied with health services increased progressively with increases in the age of patients [44].

In this study, patients who attended elementary school (grades 1 to 8) were 4.69 times more satisfied than diploma and above graduates. This study finding is consistent with studies conducted at the Kuwait capital health region [22], Jimma hospital [44], Trinidad and Tobago health centers [43], and Pakistan [38] which revealed that as educational status increased patient satisfaction decreased. However, this study is in contrast with a study done in Nekemt referral [33] and Tigray zonal hospitals [27].

At PWOPD, the availability of drugs/supplies had a statistically significant association with patient satisfaction. Accordingly, patients who did not get some ordered drugs in the hospital had their satisfaction decreased by 86% compared with patients who got all ordered drugs. This study is in agreement with studies done at Jimma University specialized hospital [31], Wolaita Sodo [30], Debrebirhan [40], and Bahirdar Felegehiwot hospitals [32]. Regarding the accessibility of health care services, the availability of latrines in PWOPD services had a statistically significant association with patient satisfaction. This study revealed that patients who were satisfied with the accessibility of latrine were 6.56 times more satisfied than their counterparts. This difference might be because of patients at PWOPD get services by their full coverage of the service payments that entitle them to accessible latrine services. When the services are inaccessible, they dissatisfied.

In this study, satisfaction with the cost of services and the availability of clean latrines had a statistically significant association in the overall model of patient satisfaction but not in the ROPD or PWOPD models. Patients satisfied with the availability of clean latrines were 3 times more satisfied than their counterparts in the overall model of patient satisfaction. This finding was in contrast with a study done in Nekemt referral hospital, which revealed that latrine related factors had no significant association with patient satisfaction in PWOPD. This observed difference might be because other services in Nekemt might have outshined and confounded latrine related factors. Patients who were satisfied with the cost of services were 2.46 times more satisfied than those dissatisfied with the cost of services in the overall model of patient satisfaction. This study was supported by a study conducted in Bangladesh and revealed that the lower the perceived overall cost of healthcare services, the higher will be the level of patient satisfaction [48]. However, this study was not in line with a study done in Hawassa teaching hospital and revealed that respondents’ rating of the amount of money they paid for services, had no statistical association with patient satisfaction [29].

### Limitations of the study

The study was not supported by qualitative methods. The findings might be subject to social desirability bias because respondents were interviewed in the hospital compound. In addition, patients might experience a relatively short memory when they feel more satisfied soon after their consultation than they do after some delayed time.

## Conclusions

Studying the factors behind patient satisfaction at regular and private wing services of the public hospitals is very important for the provision of services as per patient needs. Patient satisfaction at both regular and private wing outpatient departments was high as that of the national level. The percentage of patient satisfaction with healthcare services delivered at regular and private wing adult outpatient departments of the hospitals had no statistically significant difference.

Female sex, waiting for less than 30 min to meet services providers, got all information on drug use and their side effects, and prevention of recurrence of illness from their healthcare providers in the hospitals were positively associated with satisfaction with the regular outpatient department and the overall model of patient satisfaction.

Over 47 years of age and, diploma and above education were negatively associated with patient satisfaction at PWOPD. On the contrary, drug availability and accessibility of latrines were positively associated with patient satisfaction at PWOPD. Getting information on the prevention of recurrence of illnesses was found to be a significant predictor of regular outpatient department patient satisfaction. The availability of drugs/supplies was found to be a significant predictor of private wing OPD patient satisfaction. The availability of clean latrines and accessibility of the latrine was low in both the private wing and regular outpatient departments. Therefore, the Government investigates the implementation status of the private wing outpatient services to improve patient satisfaction. It is also better if healthcare providers explain drug use and their side effects on their patients. Hospitals should improve the availability of prescribed drugs on the premises of the hospitals.

## Supplementary information

**Additional file 1:** English version of questionnaire to assess factors of patient satisfaction in Adult Outpatient Departments of private wing and regular services in public hospitals of Addis Ababa, May 2018.

**Additional file 2:** Sociodemographic characteristics of the respondents at adult regular and private wing outpatient departments of Addis Ababa public hospitals, May 2018.

**Additional file 3:** Bi-variable and multi-variable logistic regression analysis of overall patient satisfaction at OPD of Addis Ababa public hospitals, May 2018.

## Data Availability

Data will be available upon reasonable request from the corresponding author.
