# Supplementary material for: Factors of patient satisfaction in adult outpatient departments of private wing and regular services in public hospitals of Addis Ababa, Ethiopia: a comparative cross-sectional study
Source: BMC Health Serv Res. 2019 Nov 21;19:869. doi: 10.1186/s12913-019-4685-x (PMC6873435; doi:10.1186/s12913-019-4685-x)
Supplement: Supplementary file 1 — Additional file 1: English version of questionnaire to assess factors of patient satisfaction in Adult Outpatient Departments of private wing and regular services in public hospitals of Addis Ababa, May 2018. [file 12913_2019_4685_MOESM1_ESM.doc]

**Factors of patient satisfaction in Adult Outpatient Departments of private wing and regular services in public hospitals of Addis Ababa, May 2018.**

Questionnaire No______ Hospital____________ Ward _______________

| Part 1- Household (socio-demographic and socio-economic) Characteristics | | | |
| --- | --- | --- | --- |
| Code | Questions | Responses | skip |
| 101 | What is the age of the respondent? | _____________ years |  |
| 102 | Sex | 1.Male 2.Female |  |
| 103 | Marital Status of the respondent | 1. 1.Married 2.Single 2. 3.Widowed 4. Divorced   5.Othre (specify) _______________ |  |
| 104 | Occupation of the respondent | 1. 1. Farmer 2. Government employee 2. 3. Private employee 4.Merchant 3. 5.Housewife 6.Student 4. 7.Other(specify) _______________ |  |
| 105 | What is the educational status of the respondent | Educational status………………………… |  |
| 106 | What is the religion | 1.Orthodox 2. Catholic  3.Muslim 4. Protestant  5. Other (specify) _________________ |  |
| 107 | What is the family size of the household? | Family size......... |  |
| 108 | What is the residence of the respondent | 1.Urban  2.Rural |  |
| 109 | Family monthly income | Monthly income...................... |  |
| Part 2 –Pattern of visit of OPD Pre-outpatient services and institutional service items | | | |
| No | Question |  |  |
| 201 | Do you have come to this hospital previously | 1. 1. Yes 2. 2. No | 203 |
| 202 | How can you visit this hospital? | 1. Come after referral 2. Come due to emergency 3. Come upon recommendation from friend/relative 4. Come upon personal decision 5. Other(specify) |  |
| 203 | Travel difference from home to hospital | Distance in KM.................. |  |
| 204 | Waiting time to meet service provider in minute | Time in minute........................ |  |
| 205 | Whether or not laboratory test ordered | 1. 1. Yes 2. 2. No | 207 |
| 206 | Availability of ordered laboratories in hospital | 1. 1. All in all 2. 2. Some 3. 3. Note at all |  |
| 207 | Were any X-ray /ultrasound procedure ordered for you? | 1.yes  2.no | 209 |
| 208 | Availability of ordered X-ray /ultrasound procedure in hospital | 1. All in all 2. Some 3. Note at all |  |
| 209 | What is the payment status? | 1.Free  2.Pay |  |
| 210 | Availability of sign and direction | 1.Yes  2.No |  |
| 211 | Did you have gone to drink water | 1.Yes  2.No | 213 |
| 212 | Availability of drinking water | 1.Yes  2.No |  |
| 213 | Did the provider told you how to prevent recurrence of your illness? | 1.Yes  2.No |  |
| 214 | Did the provider interview by the language you can understand? | 1.Yes  2.no |  |

Part 3: Factors regarding availability and accessibility of services. Choose from five alternatives then circle the numbers.

| No | Variable | Strongly disagree | Disagree | Neutral | Agree | Strongly agree |
| --- | --- | --- | --- | --- | --- | --- |
|  | **Accessibility & availability to health care services** | | | | | |
| 301 | How much are you satisfied with the cost paid for the services | 1 | 2 | 3 | 4 | 5 |
| 302 | Whether or not drugs /supplies ordered | 1.Yes  2.No |  |  |  | 320 |
| 303 | How much are you satisfied with the availab ility of drugs/supplies | 1 | 2 | 3 | 4 | 5 |
| 304 | How much are you satisfied with Pharmacists explain the use and side effects of medicine clearly | 1 | 2 | 3 | 4 | 5 |
|  | **Latrine related** | | | | | |
| 305 | Did you go to toilet to use latrine | 1.Yes  2.No |  |  |  | Go to other patient |
| 306 | How much are you satisfied with the access of latrine | 1 | 2 | 3 | 4 | 5 |
| 307 | How much are you satisfied with the cleanliness of latrine | 1 | 2 | 3 | 4 | 5 |

Part 4: Satisfaction of patients with different components of health care services. Choose from five alternatives then circle the numbers.

| No | Items | Strongly disagree | Disagree | Neutral | Agree | Strongly agree |
| --- | --- | --- | --- | --- | --- | --- |
|  | **Staff behavior and services** | | | | | |
| 401 | Doctor treats you very friendly and courteous manner | 1 | 2 | 3 | 4 | 5 |
| 402 | Doctors are good to explain how to prevent your disease | 1 | 2 | 3 | 4 | 5 |
| 403 | Doctors are careful to check everything when treating and examining me | 1 | 2 | 3 | 4 | 5 |
| 404 | How much are you satisfied with the information provided by doctor/nurses (courteous and respectful) | 1 | 2 | 3 | 4 | 5 |
| 405 | How much are you satisfied with the information provided by all other staffs (other than doctors and nurses) | 1 | 2 | 3 | 4 | 5 |
| 406 | How much are you satisfied with the way health providers listened to you | 1 | 2 | 3 | 4 | 5 |
| 407 | How much are you satisfied with measures taken to assure your confidentiality | 1 | 2 | 3 | 4 | 5 |
| 408 | How much are you satisfied with the overall quality of health care services in this hospital | 1 | 2 | 3 | 4 | 5 |
|  | **Physical facilities/environment** | | | | | |
| 409 | Adult OPD location is convenient for you | 1 | 2 | 3 | 4 | 5 |
| 410 | How much are you satisfied with the comfort ability of chairs in waiting area | 1 | 2 | 3 | 4 | 5 |
| 411 | How much are you satisfied with the cleanness of Waiting area | 1 | 2 | 3 | 4 | 5 |
| 412 | How much are you satisfied with the cleanliness of Examination/consultation room /OPD | 1 | 2 | 3 | 4 | 5 |
| 413 | How much are you satisfied with the overall cleanliness of the compound | 1 | 2 | 3 | 4 | 5 |
|  | **Accessibility & availability to health care services** | | | | | |
| 414 | How much are you satisfied with the waiting time to get outpatient services after registration (at waiting area) appropriateness for you | 1 | 2 | 3 | 4 | 5 |
| 415 | How much are you satisfied with time spent to get services and get back (over all waiting time) | 1 | 2 | 3 | 4 | 5 |
| 416 | How much are you satisfied with the consultation duration | 1 | 2 | 3 | 4 | 5 |
