# Supplementary material for: Factors of patient satisfaction in adult outpatient departments of private wing and regular services in public hospitals of Addis Ababa, Ethiopia: a comparative cross-sectional study
Source: BMC Health Serv Res. 2019 Nov 21;19:869. doi: 10.1186/s12913-019-4685-x (PMC6873435; doi:10.1186/s12913-019-4685-x)
Supplement: Supplementary file 2 — Additional file 2: Sociodemographic characteristics of the respondents at adult regular and private wing outpatient departments of Addis Ababa public hospitals, May 2018. [file 12913_2019_4685_MOESM2_ESM.docx]

Additional file 2: Socio-demographic characteristics of the respondents at adult regular and private wing outpatient departments of Addis Ababa public hospitals, May 2018.

| Variables | ROPD (n=488) | | PWOPD (n=467) | | Total (n=955) | | X^2^ |
| --- | --- | --- | --- | --- | --- | --- | --- |
|  | Satisfied  n (%) | Dissatisfied  n (%) | Satisfied  n (%) | Dissatisfied  n (%) | Satisfied  n (%) | Dissatisfied  n (%) |  |
| Sex |  |  |  |  |  |  | 0.29 |
| Male | 176 (83.0) | 36 (17.0) | 188 (89.1) | 23 (10.9) | 364 (86.1) | 59 (13.9) |  |
| Female | 255 (92.4) | 21 (7.6) | 234 (91.4) | 22 (8.6) | 489 (91.9) | 43 (8.1) |  |
| Age in years |  |  |  |  |  |  | 22.8^*^ |
| 18-27 | 129 (89.0) | 16 (11.0) | 77 (87.5) | 11 (12.5) | 206 (88.4) | 27 (11.6) |  |
| 28-37 | 97 (86.6) | 15 (13.4) | 87 (91.6) | 8 (8.4) | 184 (88.9) | 23 (11.1) |  |
| 38-47 | 76 (87.4) | 11 (12.6) | 83 (94.3) | 5 (5.7) | 159 (90.9) | 16 (9.1) |  |
| >=48 | 129 (89.6) | 15 (10.4) | 175 (89.3) | 21 (10.7) | 304 (89.4) | 36 (10.6) |  |
| Marital status |  |  |  |  |  |  | 5.9 |
| Single | 119 (88.1) | 16 (11.9) | 86 (87.8) | 12 (9.8) | 205 (88.0) | 28 (12.0) |  |
| Married | 272 (87.5) | 39 (12.5) | 293 (90.2) | 32 (9.8) | 565 (88.8) | 71 (11.2) |  |
| Divorced | 18 (100.0) | 0 (0.0) | 17 (100.0) | 0 (0.0) | 35 (100.0) | 0 (0.0) |  |
| Widowed | 22 (91.7) | 2 (8.3) | 26 (96.3) | 1 (3.7) | 48 (94.1) | 3 (5.9) |  |
| Occupational status |  |  |  |  |  |  | 9.5 |
| Farmer | 38 (84.4) | 7 (15.6) | 43 (97.7) | 1 (2.3) | 81 (91.0) | 8 (8.9) |  |
| Government employee | 83 (86.5) | 13 (13.5) | 84 (89.4) | 10 (10.6) | 167 (87.9) | 23 (12.1) |  |
| Private employee | 77 (87.5) | 11 (12.5) | 72 (83.7) | 14 (16.3) | 149 (85.6) | 25 (14.4) |  |
| Merchant | 34 (82.9) | 7 (17.1) | 28 (93.3) | 2 (6.7) | 62 (87.3) | 9 (12.7) |  |
| Housewife | 100 (93.5) | 7 (6.5) | 109 (94.0) | 7 (6.0) | 209 (93.7) | 14 (6.3) |  |
| Student | 52 (89.7) | 6 (10.3) | 41 (89.1) | 5 (10.9) | 93 (89.4) | 11 (10.6) |  |
| Pensioner | 20 (90.9) | 2 (9.1) | 27 (81.8) | 6 (18.2) | 47 (85.5) | 8 (14.5) |  |
| Non-employed | 13 (100) | 0 (0.0) | 10 (100) | 0 (0.0) | 23 (100.0) | 0 (0.0) |  |
| Other^1^ | 14 (77.8) | 4 (22.2) | 8 (100) | 0 (0.0) | 22 (84.6) | 4 (15.4) |  |
| Educational status |  |  |  |  |  |  | 2.9 |
| Unable to read and write | 46 (85.2) | 8 (14.8) | 58 (96.7) | 2 (3.3) | 104 (91.2) | 10 (8.8) |  |
| Able to read and write | 38 (92.7) | 3 (7.3) | 43 (89.6) | 5 (10.4) | 81 (91.0) | 8 (9.0) |  |
| Grade 1-8 | 99 (90.8) | 10 (9.2) | 101 (94.4) | 6 (5.6) | 200 (92.6) | 16 (7.4) |  |
| Grade 9-12 | 117 (89.3) | 14 (10.7) | 99 (92.5) | 8 (7.5) | 216 (90.8) | 22 (9.2) |  |
| Diploma and above | 131 (85.6) | 22 (14.4) | 121 (83.4) | 24 (16.6) | 252 (84.6) | 46 (15.4) |  |
| Religion |  |  |  |  |  |  | 3.1 |
| Orthodox | 315 (88.7) | 40 (11.3) | 309 (90.9) | 31 (9.1) | 624 (89.8) | 71 (10.2) |  |
| Protestant | 56 (84.8) | 10 (15.2) | 41 (82.0) | 9 (18) | 97 (83.6) | 19 (16.4) |  |
| Muslim | 58 (90.6) | 6 (9.4) | 68 (94.4) | 4 (5.6) | 126 (92.6) | 10 (7.4) |  |
| Catholic | 2 (100.0) | 0 (0.0) | 2 (66.7) | 1 (33.3) | 4 (80.0) | 1 (20.0) |  |
| Other^2^ | 1 (100.0) | 0 (0.0) | 2 (100.0) | 0 (0.0) | 3 (100.0) | 0 (0.0) |  |
| Residence |  |  |  |  |  |  | 0.02 |
| Urban | 380 (88.8) | 48 (11.2) | 368 (89.5) | 43 (10.5) | 748 (89.2) | 91 (10.8) |  |
| Rural | 51 (85.0) | 9 (15.0) | 54 (96.4) | 2 (3.6) | 105 (90.5) | 11 (9.5) |  |
| Family size |  |  |  |  |  |  | 7.7^*^ |
| <3 | 97 (89.0) | 12 (11.0) | 75 (88.2) | 10 (11.8) | 172 (88.7) | 22 (11.3) |  |
| 3-4 | 159 (91.4) | 15 (8.6) | 155 (92.8) | 12 (7.2) | 314 (92.1) | 27 (7.9) |  |
| 5-6 | 90 (84.9) | 16 (15.1) | 118 (88.1) | 16 (11.9) | 208 (86.7) | 32 (13.3) |  |
| >=7 | 85 (85.9) | 14 (14.1) | 74 (91.4) | 7 (8.6) | 159 (88.3) | 21 (11.7) |  |
| Hospital name |  |  |  |  |  |  | 190.3^*^ |
| St.Paul’s | 180 (89.1) | 22 (10.9) | 96 (85.7) | 16 (14.3) | 276 (87.9) | 38 (12.1) |  |
| Menelik II | 88 (88.0) | 12 (12.0) | 273 (91.9) | 24 (8.1) | 361 (90.9) | 36 (9.1) |  |
| Yekatit 12 | 163 (87.6) | 23 (12.4) | 53 (91.4) | 5 (8.6) | 216 (88.5) | 28 (11.5) |  |
| Types of OPDs |  |  |  |  |  |  | 251^*^ |
| Medical | 180 (85.7) | 30 (14.3) | 109 (93.2) | 8 (6.8) | 289 (88.4) | 38 (11.6) |  |
| Surgical | 76 (89.4) | 9 (10.6) | 32 (76.2) | 10 (23.8) | 108 (85.0) | 19 (15.0) |  |
| Guyn-Obs | 25 (92.6) | 2 (7.4) | 10 (100.0) | 0 (0.0) | 35 (94.6) | 2 (5.4) |  |
| ENT | 71 (88.8) | 9 (11.2) | 5 (62.5) | 3 (37.5) | 76 (86.4) | 12 (13.6) |  |
| Dentistry | 36 (100) | 0 (0.0) | 24 (82.8) | 5 (17.2) | 60 (92.3) | 5 (7.7) |  |
| Ophthalmology | 43 (86.0) | 7 (14.0) | 242 (92.7) | 19 (7.3) | 285 (91.6) | 26 (8.4) |  |
| Payment status |  |  |  |  |  |  | 70.4^*^ |
| Pay | 286 (88.3) | 38 (11.7) | 375 (90.1) | 41 (9.9) | 661 (89.3) | 79 (10.7) |  |
| Free | 145 (88.4) | 19 (11.6) | 47 (92.2) | 4 (7.8) | 192 (89.3) | 23 (10.7) |  |

Other^1^ = weaver, diocese, security guard, house-made, shoe-shine boy, monk

Other^2=^Jhoba witness, Wakifeta, Atheist; *P-value < 0.05 based on Chi-square test
