# Supplementary material for: Factors of patient satisfaction in adult outpatient departments of private wing and regular services in public hospitals of Addis Ababa, Ethiopia: a comparative cross-sectional study
Source: BMC Health Serv Res. 2019 Nov 21;19:869. doi: 10.1186/s12913-019-4685-x (PMC6873435; doi:10.1186/s12913-019-4685-x)
Supplement: Supplementary file 3 — Additional file 3: Bi-variable and multi-variable logistic regression analysis of overall patient satisfaction at OPD of Addis Ababa public hospitals, May 2018. [file 12913_2019_4685_MOESM3_ESM.doc]

Additional file 3: Bi-variable and multi-variable logistic regression analysis of overall patient satisfaction at OPD of Addis Ababa public hospitals, May 2018 (n=955).

|  | Variables | | ROPD (n=488) | | | | PWOPD (n=467) | | | | Total (n=955) | | | |  | | | | |  |
| --- | --- | --- | --- | --- | --- | --- | --- | --- | --- | --- | --- | --- | --- | --- | --- | --- | --- | --- | --- | --- |
|  |  |  | Satisfied n | | Dissatisfied n | | Satisfied n | | Dissatisfied n | | Satisfied n (%) | | Dissatisfied n (%) | | COR (95% CI) | | AOR (95% CI) | | |  |
| Sex | | | | | | | | | | | | | | | | | | |  | |
|  | | Male | | 176 | | 36 | | 188 | | 23 | | 364(86.1) | | 59(13.9) | | 1 | | 1 | | |
|  | | Female | | 255 | | 21 | | 234 | | 22 | | 489(91.9) | | 43(8.1) | | 1.84(1.22,2.80) ^**^ | | 2.03(1.06,3.88) ^*^ | | |
| Did the provider told you how to prevent recurrence of illness | | | | | | | | | | | | | | | | | | |  | |
|  | | Yes | | 381 | | 30 | | 366 | | 36 | | 747(92.0) | | 66(8.0) | | 3.84(2.44,6.10)^***^ | | 2.38(1.09,5.23) ^*^ | | |
|  | | No | | 50 | | 27 | | 56 | | 9 | | 106(74.6) | | 36(25.4) | |  | |  | | |
| Drug availability(n=827) | | | | | | | | | | | | | | | | | | |  | |
|  | | All in all | | 197 | | 19 | | 211 | | 8 | | 408(93.8) | | 27(6.2) | | 1 | | 1 | | |
|  | | Some | | 132 | | 26 | | 144 | | 29 | | 276(83.4) | | 55(16.6) | | 0.33(0.20,0.54)^***^ | | 0.50(0.24,1.06) | | |
|  | | Not at all | | 26 | | 5 | | 27 | | 3 | | 53(86.9) | | 8(13.1) | | 0.44(0.19,1.02) | | 0.95(0.06,15.30) | | |
| Information gained on drug use and side effects (n=827) | | | | | | | | | | | | | | | | | | |  | |
|  | | Explain all | | 216 | | 17 | | 228 | | 12 | | 444(93.9) | | 29(6.1) | | 1 | | 1 | | |
|  | | Explain some | | 116 | | 30 | | 127 | | 24 | | 243(81.8) | | 54(18.2) | | 0.29(0.18,0.47)^***^ | | 0.43(0.20,0.90)^*^ | | |
|  | | Not explain | | 26 | | 3 | | 27 | | 4 | | 53(88.3) | | 7(11.7) | | 0.47(0.19,1.12) | | 0.54(0.03,9.05) | | |
| Waiting time to enter OPD (at waiting area) in minute(n=955) | | | | | | | | | | | | | | | | | | |  | |
|  | | <=30 | | 189 | | 13 | | 161 | | 11 | | 350(93.6) | | 24(6.4) | | 1 | | 1 | | |
|  | | 31-60 | | 115 | | 10 | | 115 | | 9 | | 230(92.4) | | 19(7.6) | | 0.83(0.45,1.55) | | 0.70(0.28,1.76) | | |
|  | | 61-120 | | 64 | | 19 | | 78 | | 13 | | 142(81.6) | | 32(18.4) | | 0.30(0.17,0.54)^***^ | | 0.36(0.15,0.87)^*^ | | |
|  | | 121-180 | | 30 | | 8 | | 31 | | 6 | | 61(81.3) | | 14(21.5) | | 0.30(0.15,0.61)^**^ | | 0.41(0.13,1.28) | | |
|  | | >=181 | | 33 | | 7 | | 37 | | 6 | | 70(84.3) | | 13(15.7) | | 0.37(0.18,0.76)^**^ | | 0.42(0.13,1.34) | | |
| Satisfaction due to availability of clean latrine(n=542) | | | | | | | | | | | | | | | | | | |  | |
|  | | Dissatisfied | | 177 | | 40 | | 139 | | 32 | | 316(81.4) | | 72(18.6) | | 1 | | 1 | | |
|  | | Satisfied | | 72 | | 5 | | 74 | | 3 | | 146(94.8) | | 8(5.2) | | 4.16(1.95,8.86)^***^ | | 3.34(1.31,8.50)^*^ | | |
| Family size | | | | | | | | | | | | | | | | | | |  | |
|  | | 1-2 | | 97 | | 12 | | 75 | | 10 | | 172(88.7) | | 22(11.3) | | 1.20(0.67,2.15) | | 2.10(0.77,5.69) | | |
|  | | 3-4 | | 159 | | 15 | | 155 | | 12 | | 314(92.1) | | 27(7.9) | | 1.79(1.04,3.07)^*^ | | 1.74(0.74,4.11) | | |
|  | | 5-6 | | 90 | | 16 | | 118 | | 16 | | 208(86.7) | | 32(13.3) | | 1 | | 1 | | |
|  | | >=7 | | 85 | | 14 | | 74 | | 7 | | 159(88.3) | | 21(11.7) | | 1.17(0.65,2.10) | | 1.00(0.41,2.44) | | |
| Types of services | | | | | | | | | | | | | | | | | | |  | |
|  | | Regular | |  | |  | |  | |  | | 431(88.3) | | 57(11.7) | | 1 | | 1 | | |
|  | | Private wing | |  | |  | |  | |  | | 422(90.4) | | 45(9.6) | | 1.24(0.82,1.88) | | 1.48(0.77,2.85) | | |
| Satisfaction due to cost of services | | | | | | | | | | | | | | | | | | |  | |
|  | | Dissatisfied | | 34 | | 10 | | 39 | | 8 | | 73(80.2) | | 18(19.8) | | 1 | | 1 | | |
|  | | Satisfied | | 252 | | 28 | | 336 | | 33 | | 588(90.6) | | 61(9.4) | | 2.38(1.33,4.24) ^**^ | | 2.46(1.10,5.63) ^*^ | | |

**P -value <0.05, **P- value <=0.01, ***P- value <= 0.001, AOR: Adjusted odd ratio, COR: Crude odd ratio*
